# Unusually young onset of spastic paraparesis in a carrier of both the A431E PSEN1 and A713T APP variants

**DOI:** 10.1002/alz.088009

**Published:** 2025-01-03

**Authors:** John M Ringman, Nasim Sheikh‐Bahaei, Enrique Israel Velazquez Villareal, David Craig, Miguel Angel Macias Islas

**Affiliations:** ^1^ Department of Neurology, Keck School of Medicine at USC, Los Angeles, CA USA; ^2^ Keck School of Medicine, University of Southern California, Los Angeles, CA USA; ^3^ City of Hope, Los Angeles, CA USA; ^4^ University of Guadalajara, Guadalajara, JA Mexico

## Abstract

**Background:**

The ways in which diverse genetic variants interact to affect the phenotype of AD is poorly understood. The relatively consistent phenotype associated with specific mutations causing autosomal dominant AD (ADAD) provides the opportunity to study how other genetic variants contribute to disease manifestations.

**Method:**

We performed an in‐depth case study of a patient with the A431E *PSEN1* mutation who had onset of progressive spastic paraplegia at age 20.

**Result:**

The proband was a Mexican woman known to be at 50% risk for inheriting the A431E *PSEN1* mutation that commonly presents with cognitive changes and/or spastic paraplegia at age 40. At age 20 she began developing stiffness in her legs and difficulty walking. When seen at age 26 she had diffuse spasticity worse in the legs, slurred speech, and was cognitively intact. A flortaucipir PET scan at the time was negative as was a florbetaben scan 21 months later despite marked differences from controls in DTI measures in the corticospinal tract. Genetic screening demonstrated the presence of the A431E *PSEN1* mutation and an A713T *APP* variant which was confirmed by WGS. The A713T *APP* variant was not present in her mother who had more typical disease nor in 62 other persons of Mexican origin.

**Conclusion:**

The A713T variant in *APP* has been shown to affect APP processing and Abeta nucleation and is considered to be a risk factor for AD. We hypothesize that the presence of this variant led to the early onset of spastic paraparesis in our proband despite Abeta deposition in brain being undetectable on PET. This finding suggests spastic paraplegia may be related to aberrant *APP* processing in a manner distinct from plaque formation.

This study was funded by R01AG062007 and R01AG069013

